# Draft Genome Sequence of a Multistress-Tolerant Yeast, *Pichia kudriavzevii* NG7

**DOI:** 10.1128/genomeA.01515-17

**Published:** 2018-01-18

**Authors:** Hyun Joo Park, Hyeok-Jin Ko, Haeyoung Jeong, Sun Hee Lee, Hyun-Jun Ko, Jung-Hoon Bae, Bong Hyun Sung, Jong-In Han, Jung-Hoon Sohn

**Affiliations:** a Cell Factory Research Center, Korea Research Institute of Bioscience and Biotechnology (KRIBB), Daejeon, Republic of Korea; b Infectious Disease Research Center, Korea Research Institute of Bioscience and Biotechnology (KRIBB), Daejeon, Republic of Korea; c Department of Civil and Environmental Engineering, Korea Advanced Institute of Science and Technology (KAIST), Daejeon, Republic of Korea

## Abstract

*Pichia kudriavzevii* NG7 is a multistress-tolerant yeast, isolated from grape skins. Here, we report the draft genome sequence of *P. kudriavzevii* NG7, to understand its biochemical regulation and metabolic pathways.

## GENOME ANNOUNCEMENT

*Pichia kudriavzevii* (syn. *Issatchenkia orientalis*) has been isolated from various environments and reported to metabolize a variety of complex substrates (1, 2). This indicates that *P. kudriavzevii* is a robust yeast strain tolerant to multistress conditions (low pH, high salt and sugar concentrations, and temperature up to 45°C) (3). In particular, due to its ability to grow at extremely low pH conditions, *P. kudriavzevii* has been applied in ethanol fermentation at pH 2 and in the saccharification of lignocellulosic biomasses hydrolyzed by sulfuric acid (4–7). It also has been used in the production of succinic acid in unbuffered culture conditions (8). The use of yeast species tolerant to acid is of industrial importance for several bioproduction processes (9). Therefore, we present here the draft genome sequence of *P. kudriavzevii* NG7 as a good model yeast for multistress-tolerant species in order to understand its biochemical properties and metabolic pathways.

*P. kudriavzevii* NG7 is a multistress-tolerant yeast isolated from grape skins. Genomic DNA of NG7 was sequenced on an Illumina HiSeq 2500 platform using paired-end libraries at the Core Facility Management Center of the Korea Research Institute of Bioscience and Biotechnology (KRIBB). We used the HTQC program to eliminate low-quality (*Q* value <30) reads, generating a total of 2.5 million paired-end reads (58.7-fold coverage), before genome assembly with the SPAdes version 3.9.0 pipeline (10, 11). The genome assembly totaled 10,643,833 bp, consisting of 350 scaffolds with 39 gaps. The draft genome sequence of NG7 exhibited an overall GC content of 38.27%. The *N*_50_ value is 136,929 bp, and the length of the longest contig is 319,187 bp. Gene prediction of the genomic sequence was performed using AUGUSTUS (12). A total of 4,001 protein-encoding genes and 192 tRNA coding sequences were identified by tRNAscan-SE (13).

### Accession number(s).

The nucleotide sequence of *P. kudriavzevii* NG7 has been deposited in GenBank under the accession number NWTR00000000.
